# Pregnancy-Related Lumbopelvic Pain: Listening to Australian Women

**DOI:** 10.1155/2012/387428

**Published:** 2012-05-23

**Authors:** Heather Pierce, Caroline S. E. Homer, Hannah G. Dahlen, Jenny King

**Affiliations:** ^1^Faculty of Nursing, Midwifery and Health, University of Technology, Sydney, P.O. Box 123, Broadway, NSW 2007, Australia; ^2^Centre for Midwifery, Child and Family Health, Faculty of Nursing, Midwifery and Health, University of Technology, Sydney, P.O. Box 123, Broadway, NSW 2007, Australia; ^3^Family and Community Health Research Group, School of Nursing and Midwifery, University of Western Sydney, Locked Bag 1797 Penrith, NSW 2751, Australia; ^4^Pelvic Floor Unit, Department of Women's and Children's Health, Westmead Hospital, P.O. Box 533, Wentworthville, NSW 2145, Australia

## Abstract

*Objective*. To investigate the prevalence and nature of lumbo-pelvic pain (LPP), that is experienced by women in the lumbar and/or sacro-iliac area and/or symphysis pubis during pregnancy. *Design*. Cross-sectional, descriptive study. *Setting*. An Australian public hospital antenatal clinic. Sample population: Women in their third trimester of pregnancy. *Method*. Women were recruited to the study as they presented for their antenatal appointment. A survey collected demographic data and was used to self report LPP. A pain diagram differentiated low back, pelvic girdle or combined pain. Closed and open ended questions explored the experiences of the women. *Main Outcome Measures*. The Visual Analogue Scale and the Oswestry Disability Index (Version 2.1a). *Results*. There was a high prevalence of self reported LPP during the pregnancy (71%). An association was found between the reporting of LPP, multiparity, and a previous history of LPP. The mean intensity score for usual pain was 6/10 and four out of five women reported disability associated with the condition. Most women (71%) had reported their symptoms to their maternity carer however only a small proportion of these women received intervention. *Conclusion*. LPP is a potentially significant health issue during pregnancy.

## 1. Introduction

During pregnancy there are many discomforts experienced by women. The effects of these discomforts on the lifestyles of women are usually minor and self-limiting. Musculoskeletal complaints such as lumbopelvic pain (LPP) are described as “minor discomforts” [1, 2] or “unpleasant symptoms” [3]; however women may suffer considerable levels of pain and disability, with social and economic consequences [4]. Analgesic medications and mobility aids can be required, and life threatening conditions such as venous thrombosis have been reported as a complication of immobility [5]. LPP can impact sick leave, influence psychological health, and become a chronic pain condition [4, 6]. An increasing number of women are requesting an early induction of labour or an elective caesarean in order to achieve relief from their pain [7].

There are limited obstetric guidelines for the diagnosis and management of LPP during pregnancy. The Antenatal Care Guidelines from the National Collaborating Centre for Women's and Children's Health [8] refer to the conditions of “backache” and “symphysis pubis dysfunction” during pregnancy and recommend that more research is needed on the safety and efficacy of management strategies [9]. Over the last decade, systematic reviews [7, 10–12] have sought to bring clarification to the understanding of the conditions, and the publication of European guidelines [13] has added further to this knowledge [13]. These guidelines recommend that pelvic girdle pain (PGP) during pregnancy is a specific form of low back pain (LBP), with risk factors of a previous history of LBP and previous trauma to the pelvis. PGP can be diagnosed by pain provocation tests [14], and the recommended treatment includes adequate information, reassurance [15], and individualised exercises [16, 17].

Limited guidelines for pregnancy-related LPP may be attributable to the belief that the condition is not a serious health risk to the mother or fetus. It could also be argued that acknowledgement of pregnancy-related LPP as a pain condition creates pathology of a process that is “normal” in pregnancy, perhaps reinforcing pain catastrophising behaviour and fear avoidance beliefs [18, 19]. Guidelines for the management of nonspecific LBP in the general population emphasize that acute and chronic LBP should be viewed not just as pathoanatomical conditions, with mechanical or injury-based causes, but as conditions with psychosocial influences and consequences [20, 21]. The attitudes and beliefs of both the woman and health care practitioner shape the significance attributed to pain, clinical decision making, and recovery outcomes [22]. Whatever belief is held, pregnancy-related LPP is a condition that deserves further exploration, with translation of knowledge across countries, cultures, and health disciplines. Listening to women will add to existing knowledge and promote further understanding as to the degree of seriousness of this common complaint.

Almost half of all pregnant women and one-quarter of postpartum women are reported to experience LPP [10]. The point prevalence of pelvic girdle pain (PGP) during pregnancy is thought to be 20% [13]. The prevalence of “back pain” related to pregnancy in an Australian population has been described from population-based surveys as 35.5% and 80% [23, 24]; however the prevalence of LPP as differentiated as low back pain (LBP) and/or pelvic girdle pain (PGP) and the associated degree of pain and disability suffered by Australian women is currently unknown.

## 2. Research Aims

The aim of this research was to determine the prevalence of LPP in a sample of pregnant women attending an Australian public hospital antenatal clinic. The secondary aim was to explore the experiences of women reporting LPP, through assessment of pain and disability, differentiating low back pain (LBP) and pelvic girdle pain (PGP).

## 3. Method

A cross-sectional descriptive study was undertaken. A survey was self-administered to a cross-sectional cohort of 105 primiparous or multiparous women in their third trimester (from 28 weeks gestation) with a singleton pregnancy. Women with insufficient knowledge of the English language were excluded from the study as lack of funding did not allow for the use of interpreters and translation of the survey into other languages. Women were approached for recruitment to the study as they presented for their antenatal appointment with either a midwife or doctor. The study sample included women from medical clinics with conditions such as hypertension and diabetes. Women with inflammatory arthritis, a recent fracture or surgery to the back, hip, or pelvic area (in the previous 12 months) and/or any other serious pathology were excluded due to the possible influence on the reporting of pain. Sample size was not calculated for statistical power but selected as one manageable for the time constraints of the project.

Data were collected in the Women's Health Clinic, Westmead Hospital between 17th and 23th March, 2010. Westmead Hospital is a tertiary level hospital in Western Sydney, New South Wales, with around 4,500 births per year. The initial survey gathered data on the woman's demographics, exercise habits and lifestyle using dichotomous variable. Women who reported symptoms of LPP (LBP and/or PGP) completed a second survey including a pain diagram, Visual Analogue Scale, and the Oswestry Disability Index (version 2.1a). Closed and open-ended questions further explored the experiences of the women, for example, whether they had reported their pain to their maternity care, whether they had treatment, and whether the treatment helped. The study received the approval of Sydney West Area Health Service (Westmead Campus) Human Research Ethics Committee.

## 4. Measurement of Pain

**Figure 1:**
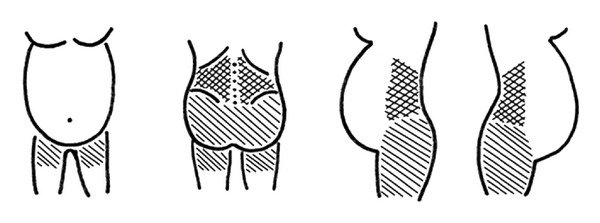
The pain diagram for self-report of LBP and/or PGP.

The pain diagram was used to self-report LPP (Figure 1). Areas marked above the level of the 5th lumbar vertebra (L5) were classified as LBP, areas marked below the level of L5 and the iliac crests (anterior, posterior, and/or lateral view) were classified as PGP, and those marked both above and below were classified as combined LBP and PGP. The Visual Analogue Scale (VAS) was used to measure pain intensity, consisting of a horizontal line, 100 mm in length, anchored by word descriptors at each end: no pain and pain as bad as it could possibly be [25, 26]. Women were asked to select the point on the scale that best represents the perceived level of pain. Pain intensity was measured for usual pain during the pregnancy and pain on the day of the survey.

## 5. Measurement of Function

At the time of data collection there were only a few tools reported for the measurement of function specifically during pregnancy [27, 28], none with proven validity [13]. The Oswestry Disability Index (ODI) (Version 2.1a) is a condition-specific tool in the management of spinal disorders that attempts to quantify the level of pain interference with physical activities by providing an estimate of disability expressed as a percentage score [29]. The index is a questionnaire with ten sections covering the assessment of pain intensity, personal care, lifting, walking, sitting, standing, sleeping, sex life, social life and travelling. The scores are calculated as a percentage; a higher percentage score indicates a greater disability. The ODI (vs2.1a) was chosen because of its use in previous studies of pregnancy-related LPP [30] and because it measures disability not just as mobility dysfunction but as a social and environmental construct. An instrument for the assessment of symptoms and activity limitation for people with PGP has recently been published [31]. This instrument is reported to have high reliability and validity both during pregnancy and postpartum and would be useful in future studies of PGP.

## 6. Analysis

The period prevalence of self-reported LPP (a retrospective recollection of pain throughout the pregnancy) and point prevalence (pain on the day of data collection) were calculated from the sample. The relationship between LPP, LBP and/or PGP and study sample characteristics was investigated. Data were analysed descriptively using PASW statistics 18, with calculation of means and standard deviations for parametric data. Pearson's Chi-Square (*X* ^²^) or Fisher's Exact Test was used to test the difference between groups for categorical, nonparametric data. The significance level was set at *P* ≤ 0.05. Segmentation of the VAS by verbal descriptors within the scale was used in subgrouping of pain level for data analysis [25]. The guidance for subgrouping of the ODI was taken from a previous study of pregnancy related LBP and PGP [30]. The Kruskal-Wallis Test tested the differences between groups for nonparametric data. Statistical methods to control for confounding variables were not employed due to the limited sample size. A thematic analysis was conducted on the open-ended question: “Is there anything else you would like to tell us about your experience of pain?” Responses were categorised according to the identification of themes and key words as written by the women.

## 7. Results

**Figure 2:**
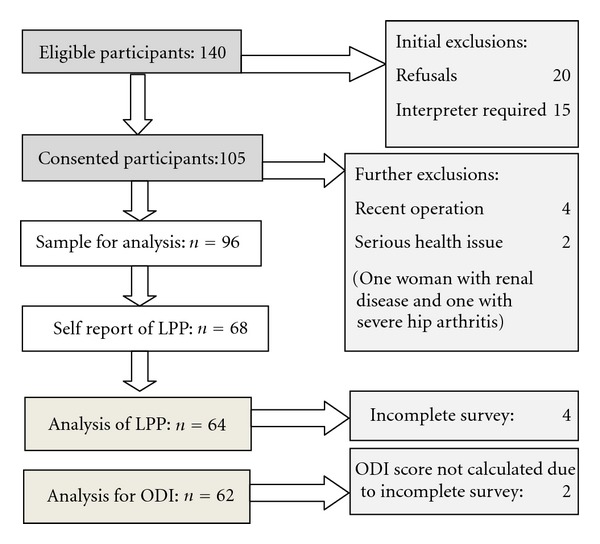
Flow chart of participants.

One hundred and forty women were approached at their antenatal appointment. One hundred and five women consented and completed the initial survey. Nine women were initially excluded due to incomplete survey (3), recent surgery (4), renal disease (1), and severe hip arthritis (1), leaving a final study sample of 96 women (Figure 2).

## 8. Study Participants

Of the 96 women in the analysis, 46 (48%) were attending a midwives' clinic and 49 (51%) a medical clinic (1 missing clinic data). Analysis of variables within the sample demonstrated no significant differences between the clinic groups in terms of age, parity, country of birth, gestation, and booking-in body mass index (BMI). The mean gestation of the sample was 34.8 weeks (range: 28–41 weeks). One-third of women were born in Australia (38.5%); 36 (37%) Asia (including 18 (20%) from India/Sri Lanka); 23 (24%) women were grouped as “other”; the largest subgroup of 6 (6%) was Middle Eastern. These data revealed a broad and reasonably representative sample of Western Sydney when compared to local demographics from the Australian Bureau of Statistics, [32] which show a high culturally and linguistically diverse population. Population characteristics as comparable to the wider geographic region of New South Wales tabled in the report: NSW Mothers and Babies, 2008 [33], are found in Table 1.

## 9. Prevalence of LPP, LBP, and/or PGP

The period prevalence of self-reported LPP during the current pregnancy was 68 (71%) and the point prevalence was 33 (34%). Of the women who reported LPP (*n* = 64) when differentiated by the pain diagram, 11 (17%) women reported LBP only, 21 (33%) reported PGP only, and 32 (50%) reported both LBP and PGP (4 excluded due to incomplete survey/pain diagram).

## 10. Risk Factors

Multiparous women were more likely to report LPP than primiparous women (*P* = 0.05). If the woman reported a past history of LPP unrelated to pregnancy, she was more likely to report LPP on the day of data collection (*P* = 0.005). Women were also more likely to report LBP (*n* = 8) or PGP (*n* = 18) if they used stairs regularly (*P* = 0.04). There was an association between regular bending and LPP reported on the day of the survey (*P* = 0.002). There was no association found between LPP and age, ethnicity, booking-in body mass index, exercise (regular, abdominal, or pelvic floor), regular lifting, or the presence of support in the home (Tables 2, 3, and 4).

## 11. Pain and Disability

**Figure 3:**
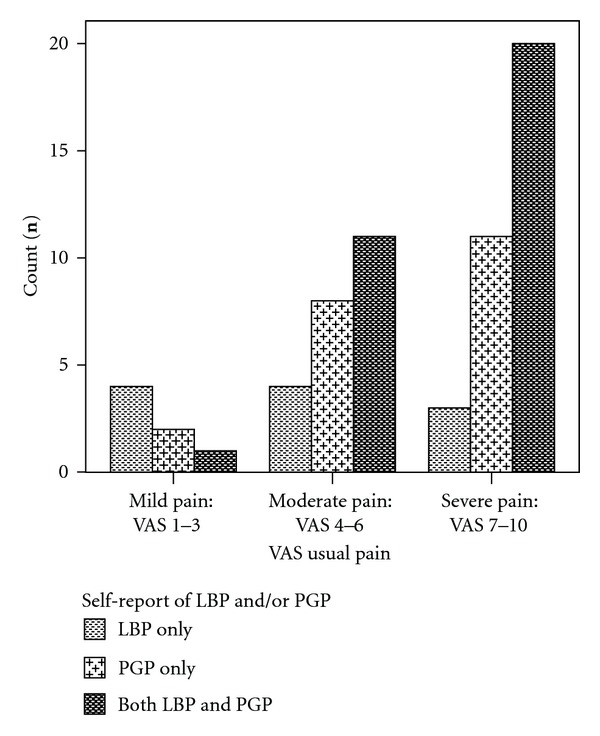
Distribution of categorised VAS scores for “usual pain” across the 3 categories: LBP, PGP, and both LBP and PGP.

The mean pain score for LPP reported by women for usual pain was 6.5 (range 1–9) and on the day of data collection was 3.8 (range 0–10). The VAS scores were subgrouped into four categories: no pain (<1), mild pain (1 to 3.9), moderate pain (4 to 6.9) and severe pain (7−10) (Figure 3). There were significant differences in pain intensity levels across the three groups: “LBP”, “PGP”, and “both LBP and PGP”, for usual pain (*P* = 0.002) and for pain today (*P* = 0.02).

**Figure 4:**
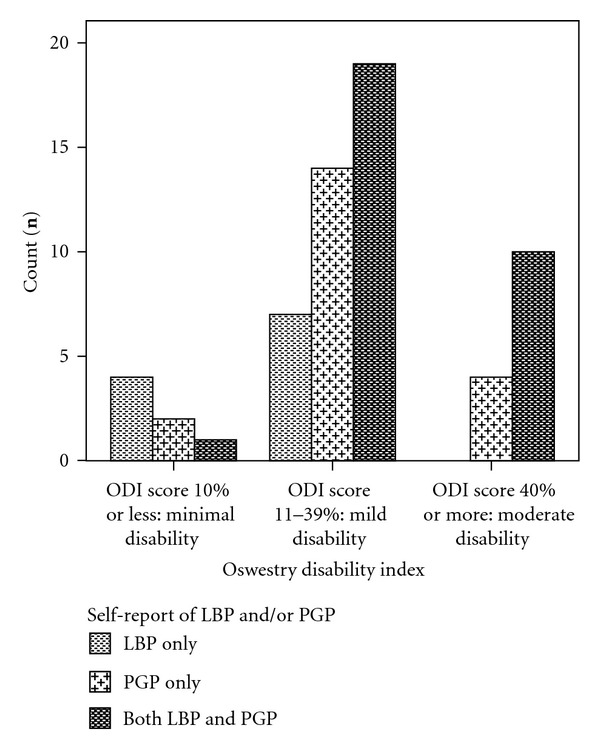
Distribution of categorised ODI scores across the 3 categories: LBP, PGP, and both LBP and PGP.

The mean Oswestry Disability Index (ODI) score for women with LPP (*n* = 62) was 29% (range: 0–74%).The ODI scores were sub-grouped into three categories: “minimal disability”: score ≤ 10%; “mild disability”: score 11 to 39%; and “moderate disability”: score ≥ 40%. Most women (*n* = 40, 65%) were classified as having a mild disability. Seven women (11%) were classified as having “minimal disability”; 14 women (23%) had a moderate disability; four of these women scored 60% or higher (Figure 4). There was a significant difference in the ODI scores across the distribution (*P* = 0.03). Women with both LBP and PGP scored a higher mean score (33.5%), therefore higher disability level than women with PGP (26%) or LBP alone (18%) (Table 5).

## 12. Listening to Women

Even though 45 (71%) of the women in the LPP sample had reported their pain to their maternity carer, only 16 women (25%) had received any form of treatment. Twelve of the women who received treatment reported benefit from the intervention. When asked why they had not received treatment, some women responded: “I was told during the last pregnancy that there was nothing that there could be done to help”; “I asked the doctor but they said it is normal in pregnancy”; “No one cared or suggested any treatment.” Other women stated: “I do not think it's necessary”; “I did not think I needed treatment”; “The pain [is] manageable”. A majority of women (70%) agreed that “LPP was to be expected because of the pregnancy.”

**Figure 5:**
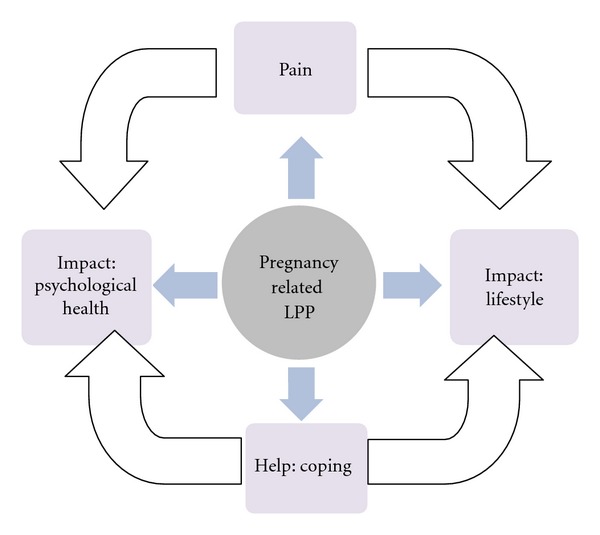
The relationship between pregnancy-related LPP and the four themes.

Eighteen women (29%) provided a response to the question: “Is there anything else you would like to say about your experience of pain?” Responses were categorised according to key words and four themes emerged from this process: pain described as a physical symptom, the impact of pain on lifestyle, the impact of pain on psychological health, and what helped the pain including coping strategies (Figure 5). Further details of the qualitative results of this study will be provided in another paper.

## 13. Discussion

The results of this study support a high prevalence of lumbo-pelvic pain (LPP) for pregnant women, both during the pregnancy (71% period prevalence) and on the day of the survey (34% point prevalence). This period prevalence is comparable to other studies which use similar definitions and a cross-sectional survey design. For example, a survey of 891 women in Sweden within 24 hours of birth reported the prevalence of LPP during pregnancy as 72% [34]; a period prevalence of 72% is calculated from a study of 213 Japanese women who were greater than 36 weeks' gestation [35]. The mean pain intensity score reported by women for this study (6.5) and the mean disability score (29%) are also similar to several other studies [34, 36, 37]. These comparable findings support this study population as a reasonably representative sample.

The importance of investigating pregnancy-related pelvic girdle pain (PGP) as distinct from low back pain (LBP) within reported LPP is supported in the literature [10, 13, 14, 30]. The conditions of LBP and PGP may coexist; however different management strategies are required for each condition [13, 38–40]. Sub-grouping of LPP also assists in identifying those women most at risk of long-term dysfunction [38, 41]. The prevalence rate of reported LBP only for this study was 17%, which is similar to that described by Gutke [30], and lower than the prevalence of PGP (33%) or combined LBP and PGP (50%) [30, 42]. When a review is made of the pain and disability levels for each of the subgroups, women with PGP only or both PGP and LBP reported higher median pain scores of 7 out of 10 for usual pain when compared to the LBP only median score of 4. These results support other research findings that LBP is less intense and less disabling during pregnancy when compared to PGP or combined pain groups [14, 30].

Many clinicians consider LPP as a normal discomfort of pregnancy. Women's comments, however, focused on a lack of acknowledgement of LPP by their maternity carer and the negative impact of pain on their lifestyle. It is apparent that for some women the pain was minimal and could be considered a discomfort, but for others, the pain was perceived as considerable. The Oswestry Disability Index attempts to measure pain interference with common daily activities. Forty women (65%) scored a mild disability (11–39%) and 14 women (23%) a moderate disability (≥40%). At least four out of five pregnant women with LPP encountered negative lifestyle consequences due to pain and disability, with one in five women experiencing a moderate level of pain-related disability. From the thematic analysis it can be hypothesized that the impact of LPP on a woman's lifestyle and psychological health is a balance between perceived pain, disability, and her capacity to elicit help and employ coping strategies (Figure 5).

Whilst most women recover from pregnancy-related LPP, some do not. Ten percent of women with pelvic pain during pregnancy still have moderate to severe pain and disability at 18 months postpartum [41]. High pain intensity scores (≥ 6 on VAS) are predictive ongoing pain and disability after birth [39, 43], and women with combined LBP and PGP recover to a lesser degree than those with PGP or LBP alone [40]. The challenge of assisting women who suffer long-term problems has been narrated in distressing case studies, including stories of surgical intervention and dramatic alterations in the lifestyles of women [44]. The complexity of chronic pain disorders drives the need for early recognition and effective management during pregnancy [40, 45].

**Figure 6:**
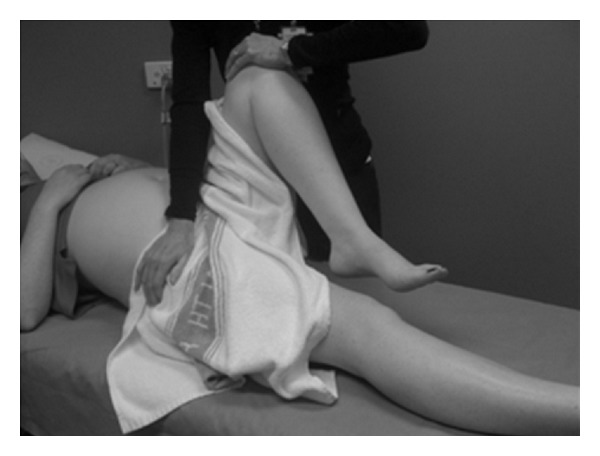
The posterior pelvic pain provocation test.

**Figure 7:**
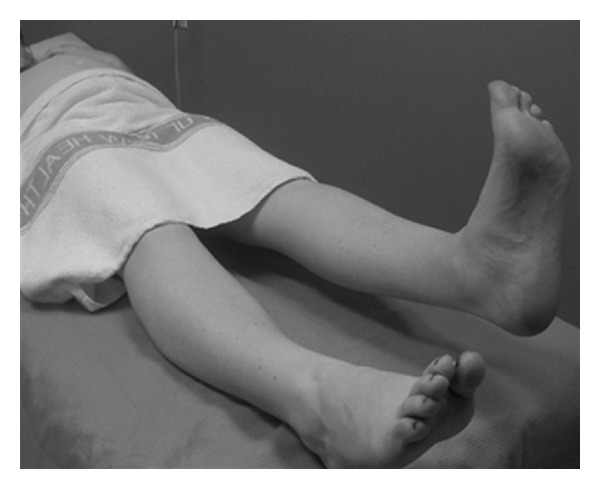
The active straight leg raise.

The identification and treatment of women at risk of chronic pain disorders could reduce the number of women with pregnancy-related LPP and impact future pregnancies [14, 35, 46]. This study supports previous findings for the identifiable risk factors for the reporting of LPP of more than one pregnancy and a previous history of LPP; however sample size lacks statistical power to make definitive conclusions. Knowledge of risk factors and aggravating activities can assist maternity carers in advising women about their condition. Objective physical assessments such as the posterior pelvic pain provocation test (see the appendix and Figure 6) have a high sensitivity and specificity [47] and can be used with pain mapping (pain diagram) to assist in diagnosing women with PGP [35, 46, 48]. The active straight leg raise (see the appendix and Figure 7) is a test of load transfer for the pelvic girdle and is predictive of pain and disability at 3 months postpartum [46].

The main limitation of this study was the sample size: as this restricted the statistical tests available for use and conclusions are therefore conservative. The authors acknowledge the possibility of bias in the study sample as women with LPP may have been more likely to agree to participate in the study. Another drawback was the need to exclude 11% of women from participating due to lack of competency in English. As previously discussed, the ODI is not a scale for pregnancy, and this limits the interpretation of the scale and the results of the study. 

## 14. Conclusion

This is the first known Australian study to report both the period and point prevalence of pelvic girdle pain as well as low back pain during pregnancy from a prospective cross sectional cohort. These results are similar to research conducted in other countries using similar methodology, however further investigation with a larger sample size is needed to provide more support to these findings.

It is recommended that low back and/or pelvic girdle pain should not be universally accepted as normal during pregnancy. In this study, the mean pain intensity score for usual pain was 6/10, and four out of five women reported disability scores with negative lifestyle consequences. Women with combined LBP and PGP, or PGP alone, experienced higher levels of pain and disability when compared to women with LBP alone. Only one-quarter of women surveyed received treatment, despite levels of pain and disability.

Future research in this area should investigate the benefit of early identification and the initiation of interventions for women with pregnancy-related LPP who are at risk of long-term problems. This may limit the development of comorbidities and chronic pain conditions. In conclusion, it would seem wise for maternity carers to listen to the concerns of women regarding pregnancy-related LPP, in order to optimise the health and lifestyle of the women in their care.

## Figures and Tables

**Table 1:** Participant characteristics.

Participant characteristic (*n* = 96)	Percentage of total sample	Percentage in NSW (2008) Total births = 94,864
Age		
<35	85.4	76.4
≥35	14.6	23.6
Parity		
Primiparous	54.2	41.6
Multiparous	45.8	55.3
BMI*		
<25	57.3	Not reported
≥25	41.7	
Country of Birth**		
Australia	38.5	69.3
Asia	37.4	12.9
Other	24	14.2

*BMI from “booking in” visit; unable to calculate BMI for one woman due to missing height/weight. **3.6% not stated in NSW report.

**Table 2:** LPP and participant characteristics.

Participant response to survey Q (*n* = 96)	LPP during pregnancy yes (*n*)	LPP during pregnancy no (*n*)	*P*	LPP on day of survey yes (*n*)	LPP on day of survey no (*n*)	*P*
Age						
<35	56	26	0.2	28	54	1.0
≥35	12	2		5	9	
Parity						
Primiparous	32	20	0.05*	17	35	0.8
Multiparous	36	8		16	28	
Ethnicity						
Australia	28	9	0.2	17	20	0.2
Asia	27	9		10	26	
Other	13	10		6	17	
BMI						
<25	37	18	0.5	18	37	0.8
≥25	30	10		14	26	
LPP in the past (unrelated to pregnancy)						
Yes	20	8	1.0	16	12	0.005**
No	48	20		17	51	

*X ^2^ (*n* = 96) = 4.7, *P* = 0.05, phi = −0.2; **X ^2^ (*n* = 96) = 9.08, *P* = 0.005, phi = 0.3.

**Table 3:** LPP, exercise habits, and lifestyle.

Participant response to survey Q (*n* = 93) (3 surveys not completed)	LPP during pregnancy yes (*n*)	LPP during pregnancy no (*n*)	*P*	LPP on day of survey yes (*n*)	LPP on day of survey no (*n*)	*P*
Regular exercise*						
Yes	39	21	0.2	18	42	0.2
No	26	7		14	19	
PF exercise*						
Yes	16	7	1.0	7	16	0.8
No	49	21		25	45	
Abdominal exercise*						
Yes	9	6	0.4	5	10	1.0
No	57	22		28	51	
Regular bending						
Yes	42	13	0.1	26	29	0.002**
No	23	15		6	32	
Regular lifting						
Yes	30	8	0.2	16	22	0.3
No	35	20		17	38	
Regular stairs						
Yes	44	19	0.8	21	42	0.6
No	22	8		12	18	
Support at home						
Yes (*n* = 91)	46	17	0.3	24	39	0.5
No	19	11		9	21	

*≥ once per week; **X ^2^ (*n* = 93) = 9.9, *P* = 0.002, phi = 0.3.

**Table 4:** LBP, PGP, or combined pain, exercise habits, and lifestyle.

Initial survey: Exercise and lifestyle	*n* = 61** (%)	LBP	PGP	Both LBP and PGP (%)	*P*
Regular exercise					
≥once per week	36 (59)	7 (70)	12 (57)	17 (57)	0.7
No regular exercise	25 (41)	3 (30)	9 (43)	13 (43)	
Regular bending					
Yes	39 (64)	7 (70)	14 (67)	18 (60)	0.8
No	22 (36)	3 (30)	7 (33)	12 (40)	
Regular lifting					
Yes	28 (45)	3 (30)	13 (62)	11 (40)	0.1
No	34 (55)	7 (70)	8 (38)	19 (62)	
Regular stairs					
Yes	43 (69)	8 (80)	18 (86)	17 (55)	0.04*
No	19 (31)	2 (20)	3 (14)	14 (45)	

*X ^2^ (*n* = 62), *P* = 0.04, phi = 0.3; **3 surveys not completed.

**Table 5:** The VAS and ODI scores for self report of LPP.

Self-report of LPP	*n* = 64 (%)	ODI % (*n* = 62)*	VAS: usual pain	VAS: pain today
		Mean (SD)	Mean (SD)	Mean (SD)
LBP only	11 (17)	18 (10.8)	4.3 (2)	2.5 (2.6)
PGP only	21 (33)	26 (15.6)	6.5 (2.2)	3.0 (2.4)
Both LBP/PGP	32 (50)	33.5 (17.4)	7.1 (1.7)	4.7 (2.7)

*Two ODI scores unable to be calculated due to incomplete survey.

## Appendix

### Physical Tests Used for PGP [13]

Tests during pregnancy should be conducted with minimal time spent in the supine position. A wedge can be provided for left lateral tilt if the woman reports supine hypotension.

Posterior Pelvic Pain Provocation Test [14] and (Figure 6)

Woman: Supine with the hips and knees flexed.
Examiner: Standing at the woman's side.
Palpate: Flex the ipsilateral hip and knee to 90°. Gently stabilise the contralateral anterior superior iliac spine with one hand.
Test: Apply a posterior force gently through the axis of the femur to the ilium thus posteriorly shearing the sacroiliac joint. Test is considered positive if pain is reproduced in sacroiliac joint or symphysis.

The Active Straight Leg Raise [49] and (Figure 7)

Woman: Supine lying, legs extended.
Examiner: Monitor the anterior superior iliac spines bilaterally.
Test: Instruct the woman to raise their leg with an extended knee (20 cm above couch). Note the ease with which they are able to do so, the provocation of any symptoms, as well as any compensatory motions of the trunk during the test. When the active (neuromuscular) system is dysfunctional, the pelvic girdle will tend to rotate towards the leg which is being raised.
